# Multiresidue Analysis of Organic UV Filters and UV Stabilizers in Fish of Common Consumption

**DOI:** 10.3390/foods9121827

**Published:** 2020-12-09

**Authors:** Sandra Gimeno-Monforte, Sarah Montesdeoca-Esponda, Zoraida Sosa-Ferrera, José Juan Santana-Rodríguez, Óscar Castro, Eva Pocurull, Francesc Borrull

**Affiliations:** 1 Instituto Universitario de Estudios Ambientales y Recursos Naturales (i-UNAT), Universidad de Las Palmas de Gran Canaria, 35017 Las Palmas de Gran Canaria, Spain; sandra32gm@gmail.com (S.G.-M.); zoraida.sosa@ulpgc.es (Z.S.-F.); josejuan.santana@ulpgc.es (J.J.S.-R.); 2 Department of Analytical Chemistry and Organic Chemistry, Universitat Rovira i Virgili, Marcel•lí Domingo s/n, Sescelades Campus, 43007 Tarragona, Spain; oscar.castro@urv.cat (Ó.C.); eva.pocurull@urv.cat (E.P.); francesc.borrull@urv.cat (F.B.)

**Keywords:** UV filters, UV stabilizers, MAE-UHPLC-MS/MS, market fish, bioaccumulation

## Abstract

Fish species can bioaccumulate different pollutants present in the marine environments and incorporate them into the trophic chain. In this work, the occurrence of organic ultraviolet (UV) stabilizers and filters in different species of fishes of high consumption has been studied. A multiresidue method based on microwave-assisted extraction and ultra-high performance liquid chromatography with mass spectrometry detection was developed and then it was applied to nine fish species from markets in the Canary Islands and Catalonia (Spain). Three UV filters (BP-3, OC and BM-DBM) and two stabilizers (UV-328 and UV-329) were found in some of the studied species, in concentrations ranging between 0.067 and 0.683 µg g^−1^ dry weight (dw). BP-3 (UV filter) was the most frequently detected compound, followed by UV-329 (UV stabilizer). Thunnus thynnus was the most heavily polluted species, with a concentration of 1.201 µg g^−1^ dw as the sum of all measured compounds.

## 1. Introduction

Research on water pollution has been commonly focused on priority compounds (e.g., pesticides and heavy metals); however, since 1990, contamination by emerging pollutants (EPs) has become a matter of growing concern due to their possible impact on the environment. These EPs not only include compounds or substances in active or past production but also their metabolites and other transformation products generated during their manufacture and use [1].

Among this large and diverse group of compounds, ultraviolet (UV) filters and stabilizer products have attracted the attention of researchers. The use of these compounds has increased in recent decades to protect against sunlight. Depending on their purpose, these compounds can be added to different consumer goods such as plastics, paints, and textiles to protect polymers and pigments against photodegradation and to prevent discolouring, or they can be used in personal care products (PCPs) to protect human skin and hair against sun damage, such as lotions, creams, and fragrances [2,3]. The input pathway of these compounds to the marine environment can be direct due to recreational activities, such as swimming or aquatic sports, but the main source is indirect, arriving through the sewage effluents from wastewater treatment plants, since the current treatment cannot completely degrade certain compounds. Furthermore, UV stabilizers are common additives of plastics, so microplastic debris are also a potential source of this kind of pollution [4]. One of the most widely used stabilizers belongs to the family of benzotriazole UV stabilizers (BUVSs).

Due to their use as additives in industry, BUVSs are listed as high production volume chemicals by the Organization for Economic Co-Operation [5], and some of them are included in the list of substances of very high concern under the EU legislation REACH (Registration, Evaluation, Authorisation and Restriction of Chemicals) [6] because of their presence in different environmental matrices and their persistent, bioaccumulative and toxic properties.

The possible effects of UV filters and BUVSs have been studied in some marine organisms such as zebrafishes (*Danio rerio*). It has been demonstrated that some UV filters can cause mortality, malformations, unsuccessful hatching, changes in lipid metabolism [7], alterations in the genes involved in their steroidogenesis and some hormonal pathways, as well as reductions in the proportion of mature spermatozoa [8]. These compounds can also cause different malformations in embryos, certain influences on neuronal and muscular development, and the behaviour in embryos [9]. Lastly, the index between the gonad mass as a portion of the total body mass of the individuals can be increased, similar to the percentage of vitellogenic oocytes in the female ovaries [10]. Regarding BUVSs, they can impact the thyroid system, stimulate some pathways related to the immune system in the brain and alter a broad number of genes that take part in the oxidative stress response [11,12].

UV filters and BUVSs are generally hydrophobic substances with a high log octanol/water partition coefficient (K_ow_) (greater than four), which makes them more likely to accumulate in sediments, suspended particulate matter and tissues of biota. In this way, these compounds have already been found worldwide in different groups of organisms: corals [13,14], molluscs [15,16,17,18,19], crustaceans [3], cetaceans [20,21] and seaweeds [22].

Although the literature about the presence of these compounds in marine fish is not extensive, they have been found in both pelagic and benthonic species [23]. UV filters and BUVSs have been measured in species from different parts of the world, such as the Antarctic area [24], the West Indies [25], Brazil [26] and different European hotspots of contamination [16]. These compounds have also been found in species with high commercial value in Europe [17] and Hong Kong [19], and in different species bought from local markets in Taiwan and Manila Bay [27,28]. Although most studies have determined the concentration in muscle as a food resource, there have been studies on BUVSs’ presence and accumulation in the liver, blood plasma, bile, brain and carcass of fishes [29].

Since the ubiquity of UV filters and BUVSs has been demonstrated in samples from human breast milk [30,31], placental tissue, urine, serum, and semen [32], it is essential to determine their occurrence in the different levels of the trophic chain.

The analysis of UV filters and BUVSs in organisms could present considerable difficulties due to the strong interaction between the compounds and the matrix studied. Therefore, achieving a selective extraction of the target compounds from the matrix is a relevant step in order to avoid the interferences that can affect their determination at the required concentrations.

Microwave assisted extraction (MAE) is an extraction technique that employs a smaller amount of the sample and shorter analysis time than those of other procedures, as well as enabling the simultaneous extraction of a larger number of samples (6–16 samples). MAE has been used for the determination of different compounds in fishes: polychlorinated biphenyls and polybrominated diphenyl ethers [33], fluoroquinolones [34], steroid hormones [35], UV stabilizers [36] and antineoplastics [37]. This technique has also been employed for the extraction of UV filters and BUVSs in solid matrices [38], in mussels [15] and in seaweed [22].

The aim of this work is the study of the presence of organic UV filters and BUVSs in fish of high consumption. For that, a multiresidue method based on MAE and ultra-high performance liquid chromatography with tandem mass spectrometry detection (MAE-UHPLC-MS/MS) was developed. The optimized method was employed to analyse seven organic UV filters and six BUVSs in nine commercial species of fishes from local markets in Las Palmas de Gran Canaria (Canary Islands) and Tarragona (Catalonia).

## 2. Materials and Methods

### 2.1. Reagents and Materials

Target UV filters and BUVSs (see characteristics in Table 1) were obtained from Sigma-Aldrich (Madrid, Spain). Stock solutions (250 µg mL^−1^) were prepared in acetone and stored in glass-stoppered bottles at 2–5 °C under dark conditions prior to use. Intermediate standards were prepared by making them from the stock solution daily in acetonitrile or methanol, depending on the purpose.

All of the liquid chromatography (LC)-grade solvents used (acetone, acetonitrile, dichloromethane, formic acid, hexane, methanol, and tetrahydrofuran) and the mass spectrometry (MS)-grade methanol used for the mobile phase were purchased from Panreac Química (Barcelona, Spain).

Phree Phospholipid Removal Solid Phase Extraction (SPE) cartridges were purchased from Phenomenex España (Madrid, Spain), and the 0.2 µm syringe polyethylene terephthalate (PET) filters from Macherey-Nagel (Dueren, Germany).

### 2.2. Sample Collection and Preparation

The different species of fishes were bought in Las Palmas de Gran Canaria (Canary Islands) and Tarragona (Catalonia). The species chosen from Las Palmas de Gran Canaria were *Scomber colias* (mackerel), *Serranus cabrilla* (comber), *Pagellus erythrinus* (common pandora) and *Sarda sarda* (Atlantic bonito), while the species bought in Tarragona were *Gadus morhua* (cod), *Solea solea* (sole), *Merluccius merluccius* (hake), *Sardina pilchardus* (sardine) and *Thunnus thynnus* (tuna). Three individuals of each species were combined as a pool in order to obtain a representative sample. Most of these species belong to the families of fish that are mainly consumed in Canary Islands and Catalonia, as well as in the rest of the country (Table 2).

The fish muscle samples, once separated from the entrails, skin, bones and head, were stored frozen at −20 °C, subject to being freeze-dried and sifted to a particle size smaller than 300 µm to obtain a powder. During the optimization process, this powder allowed a uniform contamination with the compounds and their correct extraction from the matrix.

### 2.3. Instrumentation

The lyophilisation of the samples was carried out by a LyoQuest instrument (Telstar, Barcelona, Spain). The microwave oven used for the extraction was a TITAN MPS with 16 vessels from PerkinElmer (Madrid, Spain).

The determination was carried out by an ACQUITY UHPLC (Waters Chromatography, Barcelona, Spain) equipped with a binary solvent manager (BSM) for the elution of the analytes, a 2777 autosampler and a column manager to control the temperature. It was coupled with a triple quadrupole mass spectrometry detector (TQD) with an electrospray interface (ESI). All components were controlled with MassLynx mass spectrometry software.

### 2.4. Chromatographic and Detection Conditions

For the separation, an ACQUITY UHPLC Waters BEH C18 column (50 × 2.1 mm and 1.7 µm particle size) was used at 35 °C under a flow rate of 0.4 mL min-1 for the isocratic mobile phase of methanol with 0.1% of formic acid. The ESI parameters for the mass spectrometry detection were: capillary voltage at 4 kV; cone voltage at 30 V; extractor voltage at 2 V; radio frequency (RF) lens voltage at 1 V; source and desolvation temperatures at 150 °C and 450 °C, respectively; desolvation gas flow at 500 L hr^−1^; and cone gas flow at 50 L hr^−1^. Nitrogen was used as the desolvation gas, and argon was employed as the collision gas. The detection parameters of the MS/MS for each compound are shown in the Supplementary Material (Table S1). The analysis of 13 compounds with UHPLC-MS/MS was carried out in 3 min.

### 2.5. Microwave-Assisted Extraction Optimization

The extraction process was carried out in closed vessels with 100 mg of sample. After the extraction, the vessels were allowed to cool for 10 min before being opened. The extract was filtered through a 0.45 µm syringe filter prior to being introduced into the UHPLC-MS/MS equipment.

**Figure 1:**
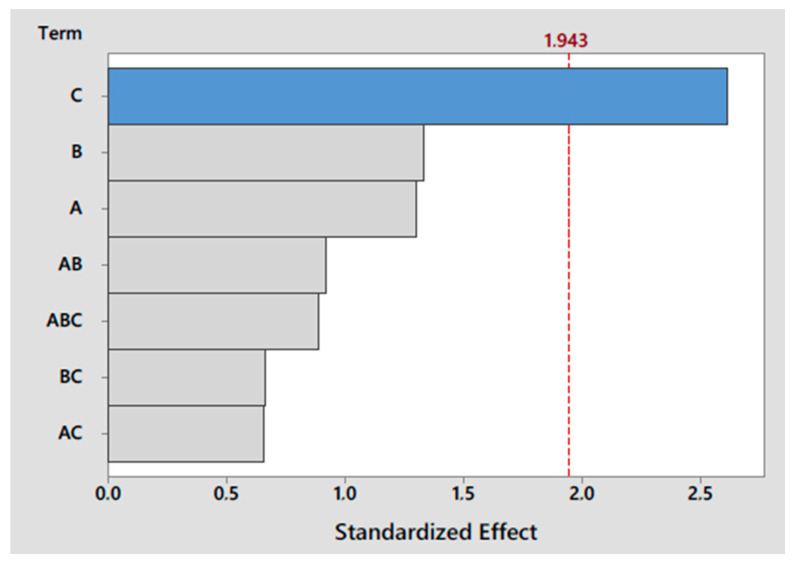
2-(benzotriazol-2-yl)-4-methylphenol (UV-P) Pareto chart of standardized effects for the factors studied in the 2^3^ experimental design (A = temperature, B = time, C = amount of sample).

The development of the MAE procedure requires the optimization of different parameters that affect the extraction efficiency. For this purpose, a mixture of fish muscle was spiked with 35 µg g^−1^ dry weight (dw) of each target compound. The variables that need to be optimized in the MAE procedure are time, temperature, amount of sample and solvent type and volume. The solvent volume was fixed in 7 mL because that is the minimum that can be used in the MAE equipment. An experimental design was necessary to choose the best conditions for the extraction, considering the influence of each variable and the correlations between them. Firstly, a 2^3^ experimental design with three variables and two levels was set: temperature (60 and 80 °C), time (5 and 10 min) and amount of sample (100 and 300 mg). The whole experimental design was carried out using 3 different extractants: methanol, acetonitrile, and a mixture 1:1 (*v*/*v*) of them. Three replicates were carried out for each experiment and the mean value was obtained. To study the relationship between these variables, a Pareto test was performed, which helps to visualize the most important variable among a set of factors (Figure 1). The amount of sample showed the highest influence on the extraction efficiency (represented with the biggest bar in the Pareto chart), while the temperature and extraction time showed less of an effect.

In examining the bivariate correlation results, with 0 being no influence, −1 maximum negative and 1 maximum positive effect, the amount of sample exhibited a strong negative influence; therefore, a fixed value of 100 mg was used, which is an advantage for the method because a low amount of sample is required. The best result regarding time extraction was obtained with the longest period explored, that is, 10 min.

**Figure 2:**
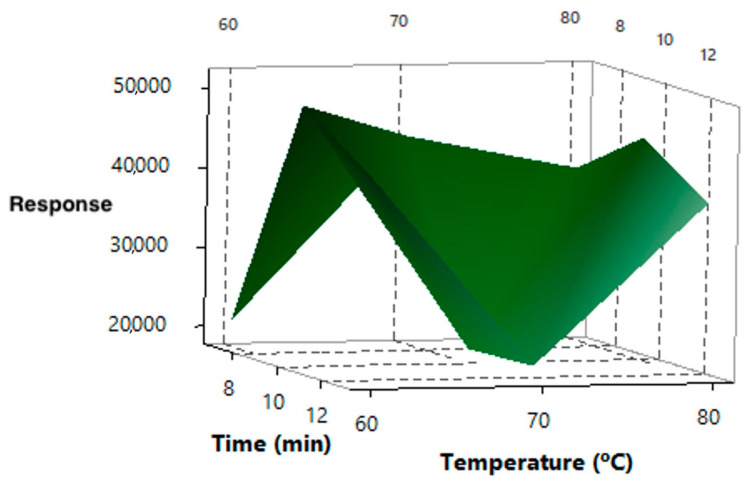
Response surface for the effect of temperature (°C) and extraction time (min) on the UV-328 extraction.

Since the effect of the temperature did not follow the same trend for all the compounds, a 3^2^ experimental design was built with two variables and three levels: 60, 70 and 80 °C, while the time of the study was extended to 7, 10, and 13 min. Acetonitrile provided the best extraction efficiencies, and it was selected as the extractant in this second experiment. As can be seen in the surface response for UV-328 in Figure 2, the best conditions were obtained with 10 min of time and 60 °C for the temperature. A similar behaviour was observed for all studied compounds.

**Figure 3:**
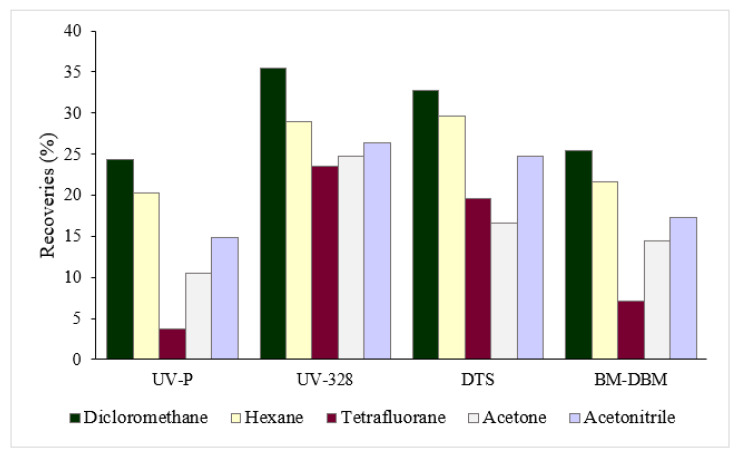
Recoveries obtained for the two benzotriazole UV stabilizers (BUVSs) and two UV filters with five different extractants. A similar trend was observed for the rest of target compounds.

Then, in order to obtain higher extraction efficiencies, other solvents more non-polar than acetonitrile were tested: acetone, dichloromethane, hexane and tetrahydrofuran. Given the characteristics of these solvents and the inability to be injected into the UHPLC system, an evaporation step was included after the phospholipid clean-up step, and the extract was reconstituted with 1 mL of acetonitrile. The highest recoveries were obtained with dichloromethane as the extractant (Figure 3).

UV-P: 2-(benzotriazol-2-yl)-4-methylphenol; UV-328: 2-(benzotriazol-2-yl)-4,6-bis(2-methylbutan-2-yl) phenol; DTS: 2-(benzotriazol-2-yl)-4-methyl-6-[2-methyl-3-[methyl-bis(trimethylsilyloxy)silyl]propyl]phenol; BM-DBM, 1-(4-tert-butylphenyl)-3-(4-methoxyphenyl)propane-1,3-dione

This methodology presents different advantages. MAE has the ability to process multiple samples at the same time under the same conditions, avoiding the occurrence of random factors that can generate differences among the samples. In comparison with the other extraction techniques commonly used with fishes, the first advantage of this methodology is that MAE uses a small amount of sample, 100 mg, compared to a weight range from 500 to 8000 mg for the other techniques used [24,28].

The second advantage is related to the volume of solvent used. During the complete procedure, only 7 mL of dichloromethane and 1 mL of acetonitrile were used. Other methodologies used to extract UV filters in fish, such as pressurized liquid extraction and ultrasonic assisted extraction, employ 25 and 60 mL of solvents, respectively [39,40]. The last advantage concerns the lack of complexity in carrying out the methodology because usually MAE does not require a complex cleaning step. In this case, because the samples were fatty tissues, only a simple phospholipid removal was required. The MAE procedure is notably easy, and it is recommendable for abundant samples in order to obtain useful results in a short period of time with a few steps and a lower expense.

In order to ascertain if the matrix effect can also cause ionic suppression during mass spectrometry detection, six non-spiked samples were subjected to the MAE procedure. After extraction, the extracts of three of them were used to check the absence of target compounds. The other three were spiked with 35 µg g^−1^ dw of each target compound. The peak areas in the spiked samples were lower than those measured in pure standard solution, which revealed the presence of the matrix effect in the ionization. The ionic suppression for the different compounds resulted in a range between 24.2 and 64.9% (Table S1).

To study the influence of the lipid content, the extraction of 12 samples spiked with 35 µg g^−1^ dw of each target compound was carried out. Then, six of them were passed through a phospholipid removal cartridge, and the other six were measured directly. The recoveries obtained using the cartridge for phospholipid removal were better for all the studied compounds; therefore, this clean-up step was implemented.

In summary, the extraction conditions were the following: 100 mg of sample was extracted with 7 mL of dichloromethane for 10 min at 60 °C. Then, the samples were passed through a phospholipid cartridge as the clean-up step and the extract was dried with nitrogen. After being reconstituted with 1 mL of acetonitrile, it was filtered again before injection, achieving a preconcentration factor of 7 times.

### 2.6. Quality Assurance

The analysis of six replicate mixture samples spiked with 35 µg g^−1^ dw of each target compound was conducted to evaluate the repeatability of the method.

Calibration curves were built for each analyte in a fish mixture using eight points between 0.07 to 14 µg g^−1^ dw, except for some of them where determination at such low concentrations was not possible, so the first point of the calibration curve was 0.5 µg g^−1^ dw for 4-MBC, UV-P, UV-326 and UV-360 or 1 µg g^−1^ dw for OC.

The limits of detection (LODs) and the limits of quantification (LOQs) were obtained from the signal/noise (S/N) response of the individual compounds from lowest point of the calibration curve, assuming minimum detectable S/N levels of 3 and 10, respectively.

## 3. Results and Discussion

### 3.1. Validation of the MAE-UHPLC-MS/MS Method

Once the determination method was optimized regarding all the involved parameters, the analytical parameters were calculated (Table 3).

The precision of the method given as relative standard deviation (RSD) was below 7% for almost all the compounds and even better for the BUVS analytes. Linear correlation coefficients higher than 0.992 were obtained from the calibration curves for the target compounds. LODs varied between 0.01 and 0.62 µg g^−1^ dw while LOQs were in the range 0.05–2.06 µg g^−1^ dw.

The results indicate that the developed method is suitable for the multiresidue determination of very varied compounds in a single and fast analysis in fish samples.

### 3.2. Presence of UV Filters and BUVSs in Market Fishes

The developed method was applied to the real samples of nine fish species bought from local markets in the Canary Islands and Catalonia (Spain).

It is important to highlight that analyte–matrix interactions are often difficult to disrupt. In this case, given the affinity of the target compounds to fat tissues, their extraction could be strongly influenced by the differences in the characteristics of the studied fish species and their variable lipid content. Among the studied species, white fish can be found (*Solea solea*, *Merluccius merluccius*, *Gadus morhua*, *Pagellus erythrinus and Serranus cabrilla*), which have barely any fat (between 0.1 and 2%) and blue fish (*Scomber colias*, *Sarda sarda*, *Sardina pilchardus and Thunnus thynnus*) containing a higher content of fat (between 8 and 15%). Moreover, it is known that their fatty acid profile and total lipid content may vary significantly depending on the species, geographical distribution and the composition or availability of the diet of the fish [41].

To overcome these differences, matrix match calibration curves were built in every single species for each analyte, in a range between 0.07 to 14 µg g^−1^ dw for most of the compounds (except for the cases previously mentioned in which higher concentrations were implemented as the first point of the curve). For each point, three triplicates were made, obtaining correlation coefficients higher than 0.990 for all the studied compounds. The repeatability was also checked in these triplicates for every concentration level of the calibration curves, built for each compound in each fish species, funding values in accordance with those obtained in the previous precision study.

Three individuals of each species bought from local markets were combined to obtain a representative sample and then the analysis of three replicates was carried out. The means of the obtained results are listed in Table 4.

Three UV filters (BP-3, OC and BM-DBM) and two BUVSs (UV-328 and UV-329) were found in the samples, with BP-3 being the most detected (51.85%) followed by UV-329 (33.33%).

BP-3 was detected in all the species except in *Pagellus erythrinus* and *Sarda sarda*. The highest concentrations were found in *Thunnus thynnus* and *Gadus morhua*, at 0.68 and 0.29 µg g^−1^ dw, respectively. UV-329 was detected in two species at high concentrations (*Thunnus thynnus* at 0.52 µg g^−1^ dw and *Sarda sarda* at 0.36 µg g^−1^ dw). UV-328 was found in three species, in *Gadus morhua* at 0.1 µg g^−1^ dw, in *Solea solea* at 0.30 µg g^−1^ dw, and in *Serranus cabrilla* but at a concentration lower than the LOQ. Although BM-DBM was detected in five of the species, the concentrations were below the LOQ.

**Figure 4:**
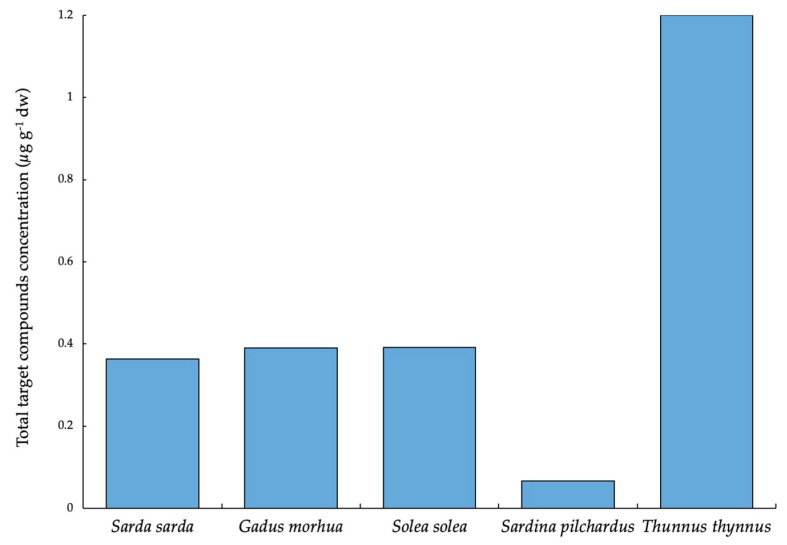
Total target UV filters and BUVSs concentration in the studied fish species.

In terms of total concentration, *Thunnus thynnus* reached the highest level of compounds, with 1.20 µg g^−1^ dw, three times greater than that of *Sarda sarda*, *Gadus morhua* and *Solea solea*. In *Merluccius merluccius*, BP-3 and BM-DBM were detected, but their concentrations were below the LOQs. Lastly, *Pagellus erythrinus* was the only one in which no compound was found (Figure 4).

These results can be compared with the study carried out by Cunha et al., in hot spots from Europe [17], such as the North-East Atlantic Ocean (Food and Agriculture organization, FAO, fishing area 27), the North Sea (FAO fishing area 27 IV) and the Mediterranean Sea (FAO fishing area 37). In that study, 4-MBC, BP-3, OC, and IMC were detected in *Scomber colias* samples, with average concentrations of 0.001, 0.016, 0.007 and 0.008 µg g^−1^ dw, respectively. In the present study, only BP-3 and BM-DBM were found, but in concentrations under the LOQs. In samples of *Solea solea*, 4-MBC was found (0.005 µg g^−1^ dw) for those authors, while in this study, only the BP-3 concentration (0.091 µg g^−1^ dw) was observed.

In *Gadus morhua*, the presence of BP-3 has been demonstrated in Taiwan, where the found concentration was 0.003 µg g^−1^ dw [28], lower than the concentration found in this study (0.294 µg g^−1^ dw). Nonetheless, in Denmark and Pacific individuals, BP-3 was not detected, but the authors have determined 0.013 µg g^−1^ dw of OC [17], which was not detected in the present study.

Regarding *Thunnus thynnus*, in the Catalonia samples, BP-3, OC and UV-329 were the compounds detected, but only BP-3 and UV-329 were in quantifiable concentrations (0.683 and 0.518 µg g^−1^ dw, respectively). Compared to the samples from the Pacific, OC has been measured (0.003 µg g^−1^ dw), while BP-3 has been found at a lower concentration (0.001 ng g^−1^ dw) [17].

Although the LODs and LOQs achieved do not allow the determination of the target UV filters at such low levels such as those obtained in other studies [17,28], it must be taken into account that a compromise between sensitivity and selectivity must be reached in order to develop a multiresidue method suitable for very varied analytes.

BUVSs have not been previously studied in the species analysed in the present work. However, in this study, a trend in UV-329 can be observed, since it was only present in the supercarnivore species of the larger sizes. This finding could be attributable to the bioaccumulation in tissues and their higher content in lipids. Regarding UV-328, it was found in two species from the Mediterranean Sea and, to a lesser extent, in one from the Atlantic Ocean.

Comparing locations, Catalonia’s species presented higher levels of compounds than the Atlantic ones, where only *Sarda sarda* showed values similar to those of the Mediterranean ones. This finding can be due to the higher production volume of EPs in the industrial area of the Mediterranean basin, and mainly in Catalonia, and because of the circulation of the Mediterranean Sea, where the water restoration time is longer.

Finally, this study enables us to compare the behaviour of some compounds depending on the species. The results show that the long-lived species that belong to the highest links in the trophic chain present higher concentrations of BP-3 and UV-329, while the concentrations of the rest of the analytes do not present this trend. The detection of these compounds can be related to other factors, such as the feeding or hatching habit or to the analytes’ characteristics; their behaviour in the aquatic system (and accumulation in the water column or in sediment) can determine the intake by organisms.

Other studies have demonstrated that the accumulation of UV filters and BUVSs in fish shows a different trend according to the family or the area where they belong, but species with similar habitats, lifestyles, and feeding or metabolic capacities have similar concentrations of these compounds [17,27]. In this way, the presence of these compounds can be determined by the different metabolic mechanisms of fishes.

## 4. Conclusions

The optimized MAE-UHPLC-MS/MS multiresidue method has allowed for the determination of thirteen compounds in different fish species in a simple and rapid way.

The differences observed in the extraction efficiencies among the target analytes can be attributed to the different structures and characteristics of the compounds and the matrix effect.

The presence of UV filters and BUVSs has been demonstrated in the fishes from markets in the Canary Islands and Catalonia. BP-3 and UV-329 were the most commonly detected and with higher concentrations. *Thunnus thynnus*, *Gadus morhua*, *Solea solea* and *Sarda sarda* were the species containing the highest total concentrations of the target analytes. Regarding the origin of the samples, the Mediterranean species presented a higher number of different compounds.

Further studies are necessary to elucidate the presence and distribution of these EPs in the trophic chain. It is necessary to not only analyse a broad range of species but also compare multiple locations and different trophic levels, which would help to understand the behaviour of the compounds depending on the species, age, habitat, feeding routines and metabolic pathways.

## Figures and Tables

**Table 1:** Characteristics of the target compounds.

Abbreviation	Common Name (IUPAC Name ^a^)	Chemical Structure	CAS Number	Log K_ow_^b^
4-MBC	4-Methylbenzylidene camphor 1,7,7-trimethyl-3-[(4-methylphenyl)methylene]-bicyclo[2.2.1]heptan-2-one	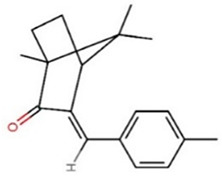	36861-47-9	4.95
BP-3	Benzophenone 3/Oxybenzone (2-hydroxy-4-methoxyphenyl)-phenylmethanone	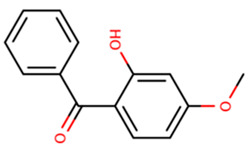	131-57-7	3.79
HMS	Homosalate (3,3,5-trimethylcyclohexyl) 2-hydroxybenzoate	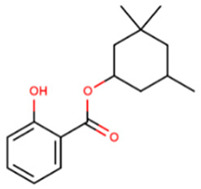	118-56-9	6.16
DTS	Drometrizole trisiloxane/Silatrizole 2-(benzotriazol-2-yl)-4-methyl-6-[2-methyl-3-[methyl-bis(trimethylsilyloxy)silyl]propyl]phenol	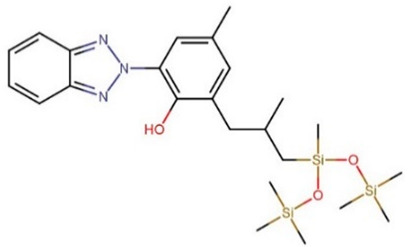	155633-54-8	10.82
OC	Octocrylene 2-ethylhexyl 2-cyano-3,3-diphenylprop-2-enoate	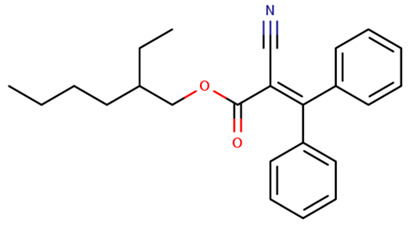	6197-30-4	6.88
BM-DBM	Avobenzone/Butyl Methoxy dibenzoylmethane 1-(4-tert-butylphenyl)-3-(4-methoxyphenyl)propane-1,3-dione	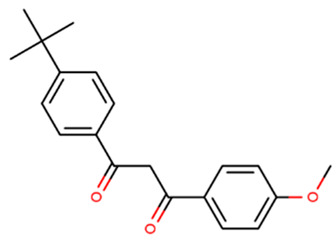	70356-09-1	4.51
IMC	Amiloxate/ Isoamyl 4-methoxycinnamate 3-methylbutyl (E)-3-(4-methoxyphenyl)prop-2-enoate	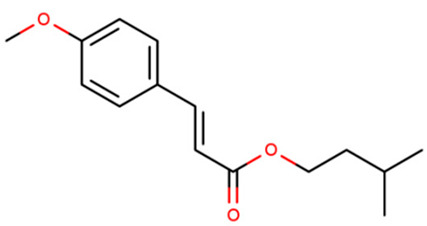	71617-10-2	4.33
UV-P	2-(benzotriazol-2-yl)-4-methylphenol	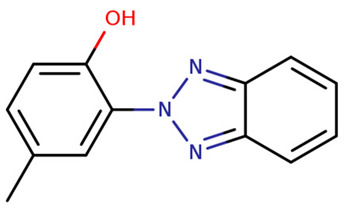	2440-22-4	2.99
UV-326	2-tert-butyl-6-(5-chlorobenzotriazol-2-yl)-4-methylphenol	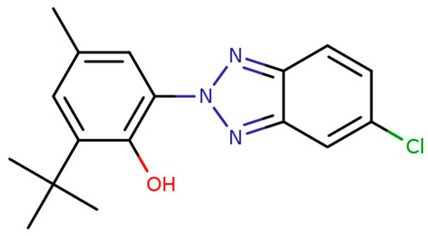	3896-11-5	5.55
UV-327	2,4-ditert-butyl-6-(5-chlorobenzotriazol-2-yl) phenol	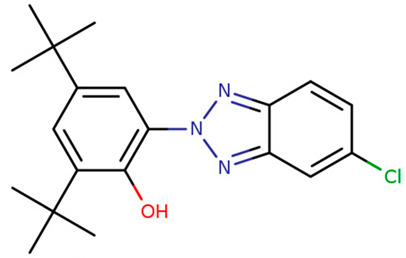	3864-99-1	6.91
UV-328	2-(benzotriazol-2-yl)-4,6-bis(2-methylbutan-2-yl) phenol	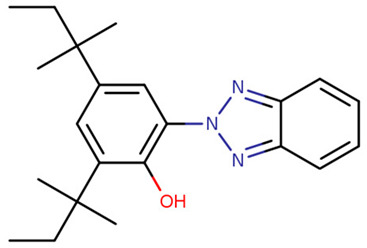	25973-55-1	7.25
UV-329	2-(benzotriazol-2-yl)-4-(2,4,4-trimethylpentan-2-yl) phenol	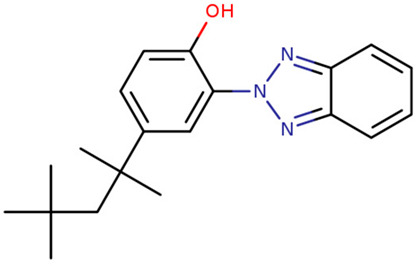	3147-75-9	6.21
UV-360	2-(benzotriazol-2-yl)-6-[[3-(benzotriazol-2-yl)-2-hydroxy-5-(2,4,4-trimethylpentan-2-yl) phenyl] methyl]-4-(2,4,4-trimethylpentan-2-yl) phenol	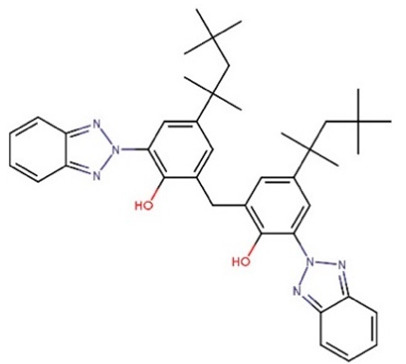	103597-45-1	12.5

^a^ IUPAC: International Union of Pure and Applied Chemistry. ^b^ Log K_ow_ was obtained from the Ecological Structure Activity Relationships (ECOSAR) Program (United States Environmental Protection Agency).

**Table 2:** Fish consumption by species in Spain, Canary Islands and Catalonia in 2019 (thousands of kg).

	Total Spain	Catalonia	Canary Islands
Total fish	555,343.34	84,841.18	18,018.60
Hake	118,151.63	19,114.53	2484.51
Sardine and anchovy	58,215.19	8827.05	644.47
Tuna and bonito	22,509.33	2478.69	2498.53
Sole	30,111.41	4477.37	961.23
Cod	41,554.10	6667.19	1441.08
Mackerel	13,760.69	2052.24	1538.56

Information obtained from Spanish Ministry of Agriculture, Fisheries and Food (https://www.mapa.gob.es/es/alimentacion/temas/consumo-tendencias/panel-de-consumo-alimentario/series-anuales/default.aspx).

**Table 3:** Analytical parameters for the developed multiresidue ultra-high performance liquid chromatography with tandem mass spectrometry detection (MAE-UHPLC-MS/MS) method.

Compound	Precision (%)	LOD (µg g^−1^ dw)	LOQ (µg g^−1^ dw)
4-MBC	7.03	0.09	0.31
BP-3	7.42	0.03	0.09
HMS	11.8	0.06	0.18
DTS	5.01	0.03	0.09
OC	6.62	0.62	2.06
BM-DBM	5.25	0.01	0.05
IMC	4.52	0.03	0.09
UV-P	1.88	0.34	1.14
UV-326	8.34	0.18	0.42
UV-327	7.78	0.04	0.15
UV-328	4.62	0.03	0.09
UV-329	3.06	0.07	0.22
UV-360	5.42	0.44	1.48

LOD: limit of detection; LOQ; limit of quantification.

**Table 4:** Concentration of target analytes (µg g^−^^1^ dry weight) measured in fish muscle samples.

Location	Fish Species	Common Name	BP-3	OC	BM-DBM	UV-328	UV-329
Canary Islands	*Scomber colias*	Mackerel	<LOQ	n.d.	<LOQ	n.d.	n.d.
Canary Islands	*Serranus cabrilla*	Comber	<LOQ	n.d.	<LOQ	<LOQ	n.d.
Canary Islands	*Pagellus erythrinus*	Common pandora	n.d.	n.d.	n.d.	n.d.	n.d.
Canary Islands	*Sarda sarda*	Atlantic bonito	n.d.	n.d.	n.d.	n.d.	0.36 ± 0.02
Catalonia	*Gadus morhua*	Cod	0.29 ± 0.02	n.d.	<LOQ	0.10 ± 0.01	<LOQ
Catalonia	*Solea solea*	Sole	0.09 ± 0.01	n.d.	<LOQ	0.30 ± 0.02	n.d.
Catalonia	*Merluccius merluccius*	Hake	<LOQ	n.d.	<LOQ	n.d.	n.d.
Catalonia	*Sardina pilchardus*	Sardine	0.07 ± 0.01	n.d.	n.d.	n.d.	n.d.
Catalonia	*Thunnus thynnus*	Tuna	0.68 ± 0.05	<LOQ	n.d.	n.d.	0.52 ± 0.02

n.d.: Not detected.

## Supplementary Materials

The following are available online at https://www.mdpi.com/2304-8158/9/12/1827/s1, Table S1: Mass spectrometer parameters for the detection of target UV filters and stabilizers.

## References

[1] Bo L., Shengen Z., Chang C. (2016). Emerging Pollutants-Part II: Treatment. Water Environ. Res..

[2] Apel C., Joerss H., Ebinghaus R. (2018). Environmental occurrence and hazard of organic UV stabilizers and UV filters in the sediment of European North and Baltic Seas. Chemosphere.

[3] Langford K.H., Reid M.J., Fjeld E., Øxnevad S., Thomas K.V. (2015). Environmental occurrence and risk of organic UV filters and stabilizers in multiple matrices in Norway. Environ. Int..

[4] Rani M., Shim W.J., Han G.M., Jang M., Song Y.K., Hong S.H. (2017). Benzotriazole-type ultraviolet stabilizers and antioxidants in plastic marine debris and their new products. Sci. Total Environ..

[5] Organisation for Economic Co-operation and Development (OECD) HPV Database Search. https://hpvchemicals.oecd.org/UI/Search.aspx.

[6] European Chemicals Agency (ECHA) Candidate List of Substances of Very High Concern for Authorisation. https://echa.europa.eu/candidate-list-table.

[7] Ziarrusta H., Mijangos L., Picart-Armada S., Irazola M., Perera-Lluna A., Usobiaga A., Prieto A., Etxebarria N., Olivares M., Zuloaga O. (2018). Non-targeted metabolomics reveals alterations in liver and plasma of gilt-head bream exposed to oxybenzone. Chemosphere.

[8] Balázs A., Krifaton C., Orosz I., Szoboszlay S., Kovács R., Csenki Z., Urbányi B., Kriszt B. (2016). Hormonal activity, cytotoxicity and developmental toxicity of UV filters. Ecotoxicol. Environ. Saf..

[9] Quintaneiro C., Teixeira B., Benedé J.L., Chisvert A., Soares A.M.V.M., Monteiro M.S. (2019). Toxicity effects of the organic UV-filter 4-Methylbenzylidene camphor in zebrafish embryos. Chemosphere.

[10] Li A.J., Law J.C.-F., Chow C.-H., Huang Y., Li K., Leung K.S.-Y. (2018). Joint Effects of Multiple UV Filters on Zebrafish Embryo Development. Environ. Sci. Technol..

[11] Li Z., Li W., Zha J., Chen H., Martyniuk C.J., Liang X. (2019). Transcriptome analysis reveals benzotriazole ultraviolet stabilizers regulate networks related to inflammation in juvenile zebrafish (Danio rerio) brain. Environ. Toxicol..

[12] Liang X., Li J., Martyniuk C.J., Wang J., Mao Y., Lu H., Zha J. (2017). Benzotriazole ultraviolet stabilizers alter the expression of the thyroid hormone pathway in zebra fish (Danio rerio) embryos. Chemosphere.

[13] Mitchelmore C.L., He K., Gonsior M., Hain E., Heyes A., Clark C., Younger R., Schmitt-Kopplin P., Feerick A., Conway A. (2019). Occurrence and distribution of UV- filters and other anthropogenic contaminants in coastal surface water, sediment, and coral tissue from Hawaii. Sci. Total Environ..

[14] Tsui M.M.P., Lam J.C.W., Ng T.Y., Ang P.O., Murphy M.B., Lam P.K.S. (2017). Occurrence, Distribution, and Fate of Organic UV Filters in Coral Communities. Environ. Sci. Technol..

[15] Bachelot M., Li Z., Munaron D., Le Gall P., Casellas C., Fenet H., Gomez E. (2012). Organic UV filter concentrations in marine mussels from French coastal regions. Sci. Total Environ..

[16] Cunha S.C., Fernandes J.O., Vallecillos L., Cano-Sancho G., Domingo J.L., Pocurull E., Borrull F., Maulvault A.L., Ferrari F., Fernandez-Tejedor M. (2015). Co-occurrence of musk fragrances and UV-filters in seafood and macroalgae collected in European hotspots. Environ. Res..

[17] Cunha S.C., Trabalón L., Jacobs S., Castro M., Fernandez-Tejedor M., Granby K., Verbeke W., Kwadijk C., Ferrari F., Robbens J. (2018). UV-filters and musk fragrances in seafood commercialized in Europe Union: Occurrence, risk and exposure assessment. Environ. Res..

[18] Nakata H., Shinohara R.I., Nakazawa Y., Isobe T., Sudaryanto A., Subramanian A., Tanabe S., Zakaria M.P., Zheng G.J., Lam P.K.S. (2012). Asia-Pacific mussel watch for emerging pollutants: Distribution of synthetic musks and benzotriazole UV stabilizers in Asian and US coastal waters. Mar. Pollut. Bull..

[19] Sang Z., Leung K.S.-Y. (2016). Environmental occurrence and ecological risk assessment of organic UV filters in marine organisms from Hong Kong coastal waters. Sci. Total Environ..

[20] Alonso M.B., Feo M.L., Corcellas C., Gago-Ferrero P., Bertozzi C.P., Marigo J., Flach L., Carolina A., Meirelles A.C.O., Carvalho V.L. (2015). Toxic heritage: Maternal transfer of pyrethroid insecticides and sunscreen agents in dolphins from Brazil. Environ. Pollut..

[21] Nakata H., Shinohara R., Murata S., Watanabe M. (2010). Detection of benzotriazole UV stabilizers in the blubber of marine mammals by gas chromatography-high resolution mass spectrometry (GC-HRMS). J. Environ. Monit..

[22] Pacheco-Juárez J., Montesdeoca-Esponda S., Torres-Padrón M.E., Sosa-Ferrera Z., Santana-Rodríguez J.J. (2019). Analysis and occurrence of benzotriazole ultraviolet stabilisers in different species of seaweed. Chemosphere.

[23] Kim J.-W., Ramaswamy B.R., Chang K.-H., Isobe T. (2011). Multiresidue analytical method for the determination of antimicrobials, preservatives, benzotriazole UV stabilizers, flame retardants and plasticizers in fish using ultra high performance liquid chromatography coupled with tandem mass spectrometry. J. Chromatogr. A.

[24] Emnet P., Gaw S., Northcott G., Storey B., Graham L. (2015). Personal care products and steroid hormones in the Antarctic coastal environment associated with two Antarctic research stations, McMurdo Station and Scott Base. Environ. Res..

[25] Horricks R.A., Tabin S.K., Edwards J.J., Lumsden J.S., Marancik D.P. (2019). Organic ultraviolet filters in nearshore waters and in the invasive lionfish (Pterois volitans) in Grenada, West Indies. PLoS ONE.

[26] Molins-Delgado D., Muñoz R., Nogueira S., Alonso M.B., Torres J.P., Malm O., Ziolli R.L., Hauser-Davis R.A., Eljarrat E., Barceló D. (2018). Occurrence of organic UV filters and metabolites in lebranche mullet (Mugil liza) from Brazil. Sci. Total Environ..

[27] Kim J.-W., Isobe T., Ramaswamy B.R., Chang K.-H., Amano A., Miller T.M., Siringan F.P., Tanabe S. (2011). Contamination and bioaccumulation of benzotriazole ultraviolet stabilizers in fish from Manila Bay, the Philippines using an ultra-fast liquid chromatography–tandem mass spectrometry. Chemosphere.

[28] Tsai D.-Y., Chen C.-L., Ding W.-H. (2014). Optimization of matrix solid-phase dispersion for the rapid determination of salicylate and benzophenone-type UV absorbing substances in marketed fish. Food Chem..

[29] Lu Z., De Silva A.O., Peart T.E., Cook C.J., Tetreault G.R. (2017). Tissue Distribution of Substituted Diphenylamine Antioxidants and Benzotriazole Ultraviolet Stabilizers in White Sucker (Catostomus commersonii) from an Urban Creek in Canada. Environ. Sci. Technol. Lett..

[30] Kim J.-W., Chang K.-H., Prudente M., Viet P.H., Takahashi S., Tanabe S., Kunisue T., Isobe T. (2019). Occurrence of benzotriazole ultraviolet stabilizers (BUVSs) in human breast milk from three Asian countries. Sci. Total Environ..

[31] Molins-Delgado D., Olmo-Campos M., Valeta-Juan G., Pleguezuelos-Hernández V., Barceló D., Díaz-Cruz M.S. (2018). Determination of UV filters in human breast milk using turbulent flow chromatography and babies’ daily intake estimation. Environ. Res..

[32] Jiménez-Díaz I., Zafra-Gómez A., Ballesteros O., Navalón A. (2014). Analytical methods for the determination of personal care products in human samples: An overview. Talanta.

[33] Wang P., Zhang Q., Wang Y., Wang T., Li X., Ding L., Jiang G. (2010). Evaluation of Soxhlet extraction, accelerated solvent extraction and microwave-assisted extraction for the determination of polychlorinated biphenyls and polybrominated diphenyl ethers in soil and fish samples. Anal. Chim. Acta.

[34] Aufartová J., Brabcová I., Torres-Padrón M.E., Solich P., Sosa-Ferrera Z., Santana-Rodríguez J.J. (2017). Determination of fluoroquinolones in fishes using microwave-assisted extraction combined with ultra-high performance liquid chromatography and fluorescence detection. J. Food Compos. Anal..

[35] Guedes-Alonso R., Sosa-Ferrera Z., Santana-Rodríguez J.J. (2017). Determination of steroid hormones in fish tissues by microwave-assisted extraction coupled to ultra-high performance liquid chromatography tandem mass spectrometry. Food Chem..

[36] Montesdeoca-Esponda S., Torres-Padrón M.E., Novák M., Krchová L., Sosa-Ferrera Z., Santana-Rodríguez J.J. (2020). Occurrence of benzotriazole UV stabilizers in coastal fishes. J. Environ. Manag..

[37] Santana-Viera S., Marzullo L., Torres Padrón M.E., Del Bubba M., Sosa-Ferrera Z., Santana-Rodríguez J.J. (2019). Microwave assisted extraction for the determination of antineoplastic compounds in marine fish. J. Food Compos. Anal..

[38] Montesdeoca-Esponda S., Álvarez-Raya C., Torres-Padrón M.E., Sosa-Ferrera Z., Santana-Rodríguez J.J. (2019). Monitoring and environmental risk assessment of benzotriazole UV stabilizers in the sewage and coastal environment of Gran Canaria (Canary Islands, Spain). J. Environ. Manag..

[39] Gago-Ferrero P., Díaz-Cruz M.S., Barceló D. (2015). UV filters bioaccumulation in fish from Iberian river basins. Sci. Total Environ..

[40] Peng X., Jin J., Wang C., Ou W., Tang C. (2015). Multi-target determination of organic ultraviolet absorbents in organism tissues by ultrasonic assisted extraction and ultra-high performance liquid chromatography-tandem mass spectrometry. J. Chromatogr. A.

[41] Ferreira I., Gomes-Bispo A., Lourenço H., Matos J., Afonso C., Cardoso C., Castanheira I., Motta C., Prates J.A.M., Bandarra N.M. (2020). The chemical composition and lipid profile of the chub mackerel (Scomber colias) show a strong seasonal dependence: Contribution to a nutritional evaluation. Biochimie.

