# Supplementary material for: Multiresidue Analysis of Organic UV Filters and UV Stabilizers in Fish of Common Consumption
Source: Foods. 2020 Dec 9;9(12):1827. doi: 10.3390/foods9121827 (PMC7763012; doi:10.3390/foods9121827)
Supplement: Supplementary file 1 [file foods-09-01827-s001.pdf]

Table S1. Mass spectrometer parameters for the detection of target UV filters and stabilizers.

Compound	Precursor ion (m/z)	Cone voltage (V)	Quantification ion (m/z)	Collision potential (V)	Confirmation ion (m/z)	Collision potential (V)	Retention time (min)	Matrix Effect (%)
4MBC	255.4	25	105	27	171	19	0.61	52.8
BP3	229.0	32	151	20	105	25	0.55	49.3
HMS	263.1	12	139	10	121	30	0.75	44.8
DTS	501.8	12	396	25	412	15	1.45	38.1
OC	362.4	28	250	12	232	20	0.52	56.8
BMDBM	311.2	30	161	23	135	23	0.65	34.9
IMC	249.1	17	161	15	179	9	0.58	36.7
UV-P	226.2	40	107	20	120	20	0.70	61.4
UV-360	658.6	40	336	25	224	35	0.91	24.2
UV-326	316.3	40	57	25	260	20	0.97	29.7
UV-327	358.3	60	57	30	302	20	0.94	29.5
UV-328	352.3	50	71	30	282	20	0.82	38.9
UV-329	324.3	50	57	25	212	25	3.11	64.9
